# Chocolate in History: Food, Medicine, Medi-Food

**DOI:** 10.3390/nu5051573

**Published:** 2013-05-14

**Authors:** Donatella Lippi

**Affiliations:** Department of Experimental and Clinical Medicine, Section of Health Services, University of Florence, Largo Brambilla 3, 50134 Florence, Italy; E-Mail: donatella.lippi@unifi.it; Tel.: +39-055-427-1826; Fax: +39-055-437-9500

**Keywords:** healthy effects of cocoa or chocolate, humoral medicine, hypochondriac melancholy, health chocolate, flavonoids, cardiovascular health

## Abstract

Throughout history, chocolate has been used to treat a wide variety of ailments, and in recent years, multiple studies have found that chocolate can have positive health effects, providing evidence to a centuries-long established use; this acknowledgement, however, did not have a straight course, having been involved in religious, medical and cultural controversies. Christian Europe, in fact, feared the exhilarating effects of new drinks, such as chocolate, coffee and tea. Therefore, these beverages would have been banished, had not doctors and scientists explained that they were good for the body. The scientific debate, which reached its peak in Florence in the 18th century, regarded the therapeutic effectiveness of the various chocolate components: it was necessary to know their properties first, in order to prepare the best cacao concoction for every patient. When Dietetics separated from Medicine, however, chocolate acquired the role of vehicle for easing the administration of bitter medicines, being associated to different health problems. The recent rediscovery of the beneficial use of cacao and chocolate focuses upon its value as supplemental nutrition. Building a bridge to the past may be helpful to detect the areas where the potential health benefits of chocolate are likely to be further explored.

## 1. Introduction

Few natural products have been claimed to successfully treat such a wide range of disorders as has chocolate, the main processed byproduct of the bean of the *Theobroma cacao* plant, as Carl Linnaeus named the “chocolate tree” in 1753 [1].

The earliest evidence for the medical use of chocolate are to be found in Mesoamerican civilizations: iconographic works and fragments, writings and remnants in the pottery suggest that cacao was prepared in beverage form at least as early as 600 B.C. There is no evidence as to whether Columbus ever tasted a drink prepared from cacao bean, which was instead well known by the Spanish conquistador, Hernán Cortés. Cortés is credited with bringing samples of cacao to King Charles of Spain in 1528, spreading the magnificent effects of the beverage prepared from this “brown gold” [2].

At the end of the 16th century, the first botanical descriptions of the chocolate tree appeared in print in Europe and the first designated shipment of cacao reached Seville, paving the way to the spreading of the use of chocolate in the Old World [3].

The medical use of chocolate in European countries, however, has at least two main reasons: first of all, chocolate was rumored to be used also by the Mesoamericans as a remedy for many disorders. Moreover, the viaticum of the medical class could give good reason for drinking a very tasty beverage, which had exhilarating effects. Christian Europe, in fact, looked to the new beverages, such as chocolate, coffee and tea, with extreme suspiciousness, and often with a condemning attitude, as they had revolutionized alimentary habits and were also supposed to upset moral behaviors, owing to their amorous properties and exciting effects. Therefore, the acknowledgement of the benefits of chocolate has not had a straight course, having been involved in religious, medical and cultural controversies [4].

## 2. The Concept of “Diet”

When chocolate started to be used in Europe, the first debate regarding cocoa product, meaning the plant and its berries and chocolate, obtained from cocoa in the form of drink, was centered on the identification of their respective qualities, in the frame of present dietetics. Dietetics was one of the three main branches of ancient medicine, and the term *diet* had a very broad meaning, encompassing all the areas that were not determined automatically by nature and that humans thus had to plan on their own. According to the teachings of classical medicine, formulated by the Greek physician Hippocrates (5th century B.C.), health was dependent on the correct balance (*eukrasía*, literally “good temperament”) of four humors: blood, phlegm, yellow bile and black bile. Their imbalance—or *dyskrasía*—was thought to jeopardize well-being and trigger disease. Diet, that is, lifestyle, had to respect several fundamental parameters that, in turn, were based on the knowledge of human nature and its temperaments and recognition of the quality of foods:

- variety: a wide range of products and the consideration of differentiating factors (meat, fruit, vegetables and cereals were assessed based on various aspects, such as provenance, climate and cultivation methods);
- personalization: activities, age, constitution, gender (children were thought to have a hot and humid nature, young people hot and dry, adults cold and dry, the elderly cold and humid; women and men also differ in nature and their regimens, thus having to envisage different substances); (1) flexibility: seasonal variables; (2) moderation: a model of ethical medicine was devised, identifying health with virtue.

In the diet of the sick, the differences in regimen were dictated also by three other factors: objectives, time and amount. The objective in this case was to restore health; timing was closely monitored by the physician and food was administered in carefully controlled quantities. The call for moderation and measure, with an eye to the close relationship between bodily health and that of the soul, had already been emphasized. Dietary conduct and behavior were regulated quite specifically, according to the humoral and allopathic tradition of Hippocratic medicine. Greater attention was paid to nutritional aspects, and the *cibus*/*potus* duo became prevalent in the concept of “diet”, as no effective therapeutic means existed at the time [5].

### Chocolate in Diet

When the use of chocolate was introduced into Western Europe, its euphoriant effects were immediately evident, to the extent that the Church stated that a religious fasting would be invalidated by drinking chocolate. It was only in 1662 that Cardinal Brancaccio declared that *liquidum non frangit jejunum* or that drinking liquid [chocolate] does not constitute a break in fasting. Meanwhile, if Europeans wanted to drink chocolate, it could only be for medicinal reasons! The concept of medical science in the 17th and 18th centuries was still strongly affected by the Hippocratic-Galenic approach, and the therapy was based on an exclusively allopathic system, by which the “hot” illnesses had to be treated with cold substances or foods, as well as “dry” illnesses needed humid foods and medicaments [4]. 

Therefore, doctors hastened to assert that chocolate was a healthy substance and used this argument to promote its pleasurable effects, consequently boosting the lucrative trade in this exotic import. From this starting point, the history of the medical use of chocolate can be reconstructed through two main kinds of sources. The first one regards the Mesoamerican tradition: the earliest authors documenting the adoption of cocoa and chocolate also for therapeutic purposes, referred to the experiences of the people in New Spain, without engaging in the analysis of the alleged reasons for these choices, but simply reporting what they had observed [2].

The second approach was centered on the identification of the qualities of cacao and chocolate, in the frame of the Hippocratic-Galenic medicine: the relationship between the balance hot/cold in the ingredients added to the medicinal preparations was dealt with in a systematic way [6].

## 3. Chocolate as Medicine: the Mesoamerican Tradition

Rumors abounded as to just how Montezuma prepared his concoction before having sexual intercourse with his numerous wives; in Mesoamerica, cacao was prepared only as a beverage. It was a very precious substance and, therefore, reserved for priests, highest government officials, military officers and great warriors, and it was supposed to be unsuitable for women and children [2].

The first Europeans to encounter cacao were travelers. In 1601, the Florentine businessman and circumnavigator, Francesco d’Antonio Carletti, submitted his report to Ferdinando I de’ Medici, Grand Duke of Tuscany: Carletti wrote a detailed description of the cacao growing and chocolate making processes and stated that chocolate was a very strengthening mixture prepared using cacao and water [7].

The Spanish monk, Bernardino de Sahagún, was the extensor of the Florentine Codex (dated to 1590): he left Spain for New Spain in 1529 and, for the next 60 years, collected extensive information on Mexican agriculture, botany, cultural practices, dietary patterns and health and medical practices. Bernardino de Sahagún, for instance, related the use indigenous Americans made of beverages prepared with cocoa for therapeutic purposes, depending on the type and dosage; he warned against excessive drinking of cocoa prepared from unroasted beans, but recommended it if used in moderation. He maintained that drinking large quantities of green cocoa made imbibers confused and deranged, but if taken in moderation, the beverage was invigorating and refreshing [8].

A second primary source for information on Mexican medicinal use of cacao is the Badianus Manuscript (dated to 1552), which includes remarkable paintings of medicinal plants and a rich text that provided a critical understanding of Mexican disease, nutritional problems and healing techniques.

The author of the Badianus Manuscript, M. De La Cruz, was a Mexican teacher at the College of Santa Cruz, founded by the Spanish around 1536 in Mexico City: the manuscript presents Mexican disease concepts and treatments, underlining a clear prevalence of the use of cocoa derivatives as nutrients or remedies for angina, constipation, tartar-related dental problems, dysentery, dyspepsia, indigestion, fatigue, gout and hemorrhoids [9].

If Friar Agustín Dávila Padilla reported its use in curing kidney disease, it is Francisco Hernández, in 1577 who identified some fields in which cocoa was profitably used as medicine [10].

Hernández (*c*. 1517–1587) was arguably one of the most important European physician-botanists of the 16th century: he identified and described more than three thousand plants that were not known in Europe [11].

## 4. Chocolate as Medicine: the European Tradition

In 1570, Hernández was sent by Philip II to New Spain, where he would lead a scientific expedition to study native flora, gathering information from the natives about herbs, trees and medicinal plants to learn their uses, doses, as well as the conditions for cultivation, so that medicinal plants could be grown in Spain. Hernández’s monumental work was never published in his lifetime, and the manuscript that Philip II had deposited in the library of the Escorial was burnt in the terrible fire of 1671. The Accademia de Lincei edition of Hernandez’s Mexican Treasury, published in 1651, is often referred to as “The Rome Edition”; this was due to Cassiano dal Pozzo (1588–1657), a seventeenth century patron of the arts and sciences and famous antiquarian, who became a member of the Accademia dei Lincei. When he visited the Escorial Library, he asked the principal archivist and librarian, Andrés de los Reyes, to copy several important works by Hernández, among which is the Index Alphabeticus Plantarum Novae Hispaniae, which was used for the Rome Edition and is of great importance in reconstructing the works of Hernández burned in the 1671 fire [12].

Hernandez was among the first to consider the use of chocolate in connection with humoral medicine: he argued that as cacao bean had cold and dry qualities, chocolate had to be recommended to treat hot diseases, such as fevers. Hernandez reported the different prescriptions followed by natives: without adding any other ingredients, chocolate was used for liver disorders; four grains of cocoa and a dose of gum (*holli*) toasted and mixed together “restrained dysentery”; the drug called *atextli* was made using a fine paste of cocoa and corn, to which *mecaxochitl* (*Piper sanctum*) and *tlilxochitl* (*Vanilla planifolia*) could be added, as an aphrodisiac. The frequent use of a beverage made with grains of *pochotl* and *cacahoatl*, instead, was extremely fattening and was therefore recommended to slim and asthenic people [4].

However, the medical use of cocoa needed to be tested within the framework of the European medical understanding of the time.

Santiago de Valverde Turices discriminated the cold quality of cocoa from the hot and dry qualities of chocolate, which had then to be used for the cold and humid illnesses: useful for breast illnesses if drunk in large quantities, it could also be beneficial for the stomach if taken in small doses. When people were healthy, the use of chocolate was subordinated to the addition of “cold” ingredients to balance its “hot” nature [13].

Spanish doctors were very active in this debate; in 1618, Bartolomeo Marradon wrote a dialogue, which summarized the different uses of cocoa in Spain: cocoa was used as currency or as beverage, but it could be employed appropriately as medicine, as “panecitos, tabillas or en caxa como conserva”. This cold drink did not cause drunkenness and it could be prepared in different ways, adding different ingredients, suitable to the pathology that had to be cured. Mixed with sugar, it could be given to the ill “cuando no hay calenture”. Cinnamon, sugar, pepper, cloves, vanilla and anises were the ingredients that were included, as variations, in Marradon’s recipe, which provided this prescription: “700 (grains of) cocoa, 1 and ½ pounds of white sugar, 2 ounces of cinnamon; 4 grains of Mexico powder, called chili or pimiento; ½ ounce of cloves; 3 little rinds of Campeca (or, instead of this, the weight of 2 realil of anise); in the end, 1 hint of “achiote” enough to give it color. Some add almonds, knuckles and Orange flowers’ water”. Anyway, Marradon did not overlook the negative features of chocolate, castigating it as potentially ‘obstructive’ [14].

For this latest statement, Bartholomeo Marradon became Colmenero de Ledesma’s target in his book: first published in Madrid in 1631 under the title *Curioso tratado de la naturaleza y calidad del chocolate*, Colmenero de Ledesma’s work was subsequently reprinted in many editions and translated versions. Colmenero de Ledesma fundamentally disagreed with Marradon, arguing that chocolate was a beneficial health drink and could fit in with his Galenic medical philosophy. 

Colmenero de Ledesma was a physician from Andalusia, as made plain in the title page of his book; this detail, in conjunction with commendations from some of the leading medical practitioners of his day—Dr. Mechor de Lara, the Physician General of Spain and Dr. Juan de Mena, the Physician to the King of Spain—point to the medical nature of Colmenero de Ledesma’s work. The Author concentrates his work on answering four questions: what is chocolate and what are the qualities of cacao; what is the quality of chocolate; how to make it and how to drink it; when to drink it and how much. Colmenero de Ledesma provided a recipe for drinking chocolate, which shows how Spanish consumers were gradually changing the flavor of the import by adding sugar and spices more congenial to their palate than the rather more bitter taste favored by the natives. According to him, chocolate was a *medicinal* drink, with specific health-giving qualities. [15].

In this, he was not alone: the list of the accounts that, from the 17th through to the 19th centuries, referred to the presumed merits and medicinal properties of cacao and chocolate, is infinite and can be divided into three main groups: (a) to enable patients to gain weight; (b) to stimulate nervous systems; (c) to improve digestion [1].

The geographical distribution of chocolate supporters mirrors the trend of the economic interests of the different countries. Anglo-Saxon authors, in fact, took part in this debate at the time when Great Britain undertook the conquering of the new continent. Henry Stubbe, Charles the II’s doctor and philosopher Thomas Hobbes’ friend, starting from the analysis of the previous authors’ testimonies, wrote his monograph in 1662 to warn his readers on chocolate-related mistaken beliefs. Stubbe dedicated his treatise to his famous colleague Thomas Willis. Willis, in fact, had recommended chocolate to prevent apoplexy: he prescribed a mixture of chocolate, which contained the powder of “the root of the male peony”, mixed with human skull, ambergris and musk. Stubbe made a long list of the medical uses of chocolate, which was considered suitable to cure also the “hypochondriac melancholy”, caused by vein obstruction by black bile, accompanied by stomach weakness and weight loss: in this affirmation the reason of the dedication to Willis, pioneer of brain research and nervous diseases, is likely to reside [16].

In the same years, the Roman physician, Paolo Zacchia, prescribed chocolate as a treatment for hypochondria [17].

According to Stubbe’s observations, the use of chocolate seemed extremely positive as expectorant, diuretic and aphrodisiac. He emphasized how, in the Indies, chocolate was drunk under the doctor’s prescription once or twice a day and that it was particularly useful to restore energy “if one is tired through business and wants speedy refreshment”*.* Stubbe not only told about the use of chocolate in the various sources, but proposed his own recipe, which closely resembles the one, referred to by Colmenero de Ledesma: “For every 100 cocoa seeds, 2 hot red peppers (*chile*), a handful of anise seeds and *orichelas* (*orejaelas*) and 2 of flowers called *mecasuchill*, 1 vanilla or 6 powdered alexandrine roses, 2 drams of cinnamon, 12 almonds and as many nuts, ½ pound of sugar and as much *achiote* to give color”. In reference to cocoa nutritional value, Stubbe noticed how the British soldiers stationed in Jamaica, where many plantations had been organized, lived primarily on cocoa paste, mixed with sugar, which was then melted in water; again, on future occasions, chocolate and cocoa were commonly used by the armies as one of the main means of livelihood and solace [16].

Most probably his most important source was Dr. Francisco Ferdinandez, a prominent personality in Philip the II Mexico’s court, to whom William Hughes also referred in his ethno-botanic work on plants that grew in the English plantations in America in 1672 [18].

However, during the 17th century and in the first half of the 18th, the question about the medical use of chocolate entered the Academy, and it was also the subject of some degree theses at the medical Faculty of Paris, between 1684 and 1736. Particularly significant, in this perspective, seems to be Franciscus Foucault’s dissertation, discussed in 1684 at the medical Faculty of Paris, under M. Stephanus Bachot’s presidency: after a brief introduction on the different kinds of foods, the use of chocolate is described. The preparation methods were different, just as the forms in which it could be packed, but chocolate taste was always so pleasant that the author concluded, almost in a lapidary way: “Therefore, the use of chocolate is salubrious [for] it excites and strengthens with its warm mild juiciness the bowel’s inborn warmth and strength, it helps digestion, it fosters the spread of food and the secretion of the unnecessary, it accumulates fat, it is not an enemy to the brain, it is Venus’s friend and very suitable for body and soul” [19].

Despite these enthusiastic statements about the medical use of chocolate, not all the doctors agreed on its beneficial effects, and its detractors accused it of causing major health problems: in particular, this debate, between the favorable and the opposed, started in Florence in the early 18th century, where only in 1728, four pamphlets were published, which vouch for this controversy.

### In Florence

The news about chocolate officially got in Florence starting from the end of the 16th century; the custom of drinking chocolate “after the use of Spain”, however, dated back to 1668, according to the words of two doctors of the time, Giovanni Targioni Tozzetti and Antonio Cocchi: “(…) it has been introduced in Florence this year, 1668, commonly a beverage to the use of Spain, called Chocolate, and one of the Storekeepers aforesaid sells it in little clay cups, and it seems to be tasty be it hot, or cold” [20].

According to a writer of the 19^th^ century, the first one to manipulate and sell chocolate in Florence as a heart medicament was an apothecary, named Tozzetti, at the time of the Grand Duke Ferdinando I de’ Medici [21].

The Medici Grand Dukes and their family were fond of chocolate: in a letter written by Father Ettore Ghislieri to Cardinal Leopoldo de' Medici in 1671, the former thanks the Cardinal for a box of assorted chocolates, mentioning his favorite varieties as a citron-flavored one and one based on a Spanish recipe. He also states that his doctors have endorsed chocolate as a treatment for his flatulence [22].

The Medici court fostered both scientific rigor and unbridled gluttony, and Francesco Redi, doctor and scientist at the Medici court, balanced both with tact and humor: despite his affirmation in his poem, *Bacco in Toscana* (*That chocolate might not yet/be taken up or even tea, /medicines like this/ would never do for me*), his name is firmly bound to chocolate for his having invented the recipe of a special jasmine-smelling chocolate, then patented in a formula, which listed ingredients, amount and procedure, under the name “Grand Duke’s recipe”. This recipe, however, remained a state secret till 1712, as the Grand Duke had prohibited to reveal it in writing: it was possible to sip this drink only in Florence and, even here, only at the Court or in the houses of families of the highest nobility [23].

In Florence, chocolate was really appreciated, influencing also market development. In the 18th century, the scientific controversy catalyzed between two peculiar personages: Doctor Giovan Battista Felici, great chocolate accuser, who considered it “disorder (...) to shorten life”, brought by “men’s intemperance”, and Francesco Zeti, nicknamed “the Hunchback of Panone” after the coffee-house where he worked, who was worried that all this “talking bad” about chocolate could provoke a decrease of customers and, fearing to be discharged in his turn, wrote a short book in defense of chocolate. 

Cocoa, according to Felici, could not be considered a cold substance, since plants take their qualities from the places in which they grow and, therefore, “it contains a loose, fat and viscous substance, which can easily contain the particles of the heat”: the presence of “oil” and its “bitter taste” proved it. It is for this reason that, according to Felici, the custom spread to add some particular flavors in the composition of chocolate, such as cinnamon, vanilla, pepper, “cloves, amber and *acciotte* and other similar very hot spices”. Chocolate has then a sort of “slow, long-lasting fire”, causing in the blood “significant fermentation, which can spoil it”, since “it causes an extraordinary motion in the animal instincts; so, when it gets into the stomach, it makes us more able to perform our doings with vivacity” [24].

In evaluating the nature of the various substances in the medical writings of this period, the indirect contribution of increasing iatrochemistry is evident: with the suggestions generating from chemistry, the consequences of the use of chocolate, even in the decomposition of the different parts of the blood, were hypothesized, either for the “viscous nature of cocoa, able to enlarge excessively the body fluids”, preventing them from circulating or for the “obstructions” caused by the impossibility of chocolate to spread “into all part of the body”, again for its viscosity. Heart palpitations, intermittent pulse, convulsive movements and apoplexy could be induced by the use of chocolate, and also the ingredients with which it was mixed, such as cinnamon and vanilla, contributed to its negative effects, as they contain “irritant, volatile and stinging substances, which can spoil in thousands of different ways the natural composition of the body fluids”, affecting also the nerves and, as a consequence, the “animal instinct”: equally negative effects were ascribed to sugar, and the use that painters made of it to blend the colors demonstrated its viscosity. Damages to the fluids and damages to the solid parts of the body were hypothesized: adhering to the inner membrane of the stomach, chocolate inhibited its gastric juices, corrupting the nerves “texture” as a result of its “constant contractions”. The muscles were also affected by this excessive contractile activity and the heart itself suffered: the cases in which the use of chocolate could be beneficial were hemorrhages, as proven by the fact that “Florentine ladies (...) where healed from the copious blood loss, by the continuous use of chocolate, which they used as desiccant and astringent medicament” [24].

These were the faults according to Doctor Felici: Francesco Zeti’s retort was published the following year. He claimed to have commissioned from an anonymous doctor (maybe Girolamo Giuntini) a sort of defense of chocolate to protect his own interests: regardless of its alleged quality of hot or cold substance, cocoa was not “replete of oleaginous and sulfurous parts”, since it owns also a “milky spirit”, which could have beneficial effects on the human body. The fact that the cocoa plant grows in warm places could not be a support to its quality, since there exist numerous plants, which disprove this relationship [25].

At this point, the pharmaceutical factories were involved in this debate, which were particularly active in cocoa processing, elaborating personalized recipes, even for cosmetic purposes. Among them, the Grand Ducal Spezieria and the Officina Farmaceutica in Santa Maria Novella was particularly dynamic: right after an inspection at Santa Maria Novella drugstore on November 23, 1751, by a medical committee in which the doctor Giovanni Targioni Tozzetti also participated, chocolate was served in silver bowls in the Green Room [26].

Different views were summarized, a few years later, by Giovan Battista Anfossi, who examined in a very detailed way the various authors’ opinions, referring most of all to the English authors, like Stubbe, who represented an original voice in this debate, coming from a different cultural environment, compared to the Spanish speaking authors. Through the lines of his dissertation, in addition, new issues came up, compared to previous authors: enumerating the criticism of which chocolate was object, in fact, Anfossi did not limit himself to mention the “stubborn Galenics”, who based their beliefs on the qualities of the various substances, but he referred to the “Professors of more reasonable Systems”. It is interesting to note that Anfossi referred to chocolate implementation even for a topical use, as cocoa “butter”, unequivocally solving the riddle about the use of chocolate in curing hemorrhoids, which was transmitted by previous authors, apparently colliding with the most elementary precautions to be taken to avoid the annoying inconvenience: “If hemorrhoids are aching and inflamed, the basic bleeding is convenient … sweet oil, simple, butter or cocoa, butter…” [27].

A special analeptic chocolate was sold in Florence in the middle of the 19th century: cocoa of Caracca, cinnamon of Ceylon and sugar of Avana or “potato flour”. It could be mixed with milk, cream and coffee spirit, to obtain a very enjoyable drink. It was recommended for those people who suffered from weakness and chronic lung catarrh and for the victims of serious acute ailments [28].

## 5. Last Chapters

The 18th century counts many other statements on the medical use of cocoa and chocolate, not the last of which is the one from Carl von Linné (Linnaeus), in which are summarized chocolate’s qualities as nourishment and as therapeutic substance. Linnaeus identified three kinds of illnesses in which chocolate could be used appropriately: loss of weight, as a consequence of lung and muscles diseases, hypochondria and hemorrhoids, adding also that it was an excellent aphrodisiac, confirming a tradition already existing in the Pre-Columbian culture [1].

Later, in Blancardi’s work, it is stated that “Chocolate is prepared with milk or water; when shaken, it melts and it must be drunk hot. It is a nutrient beverage, useful to the old and to the weak.” [29].

After Lardizabal and Buchan’s works, the century ended with Antonio Lavedan’s work, who claimed, again on the basis of the previous tradition, that chocolate was a sort of universal medicine, since it stimulates natural warmth and the heart, decreasing flatulence, resolving constipations, helping digestion and appetite, increasing virility and slowing down white hair growth, prolonging lifetime significantly [30,31,32].

The history of the medical use of chocolate had reached its peak. In the new form of a solid bar, the enduring reputation of chocolate as both a medicine and a food improved even more, and its duplicity held sway for years within the advertising market. At the same time, a new chapter in the history of chocolate for a therapeutic use started, destined to maintain mainly its leading role of excipient, to mask the bitter taste of medicines: under the name *health chocolate*, a wide range of drug concoctions used chocolate or cocoa nomenclature to help promote their sales, and entire lines of chocolate-flavored medical products were produced. The 19th century experienced, therefore, a great increase in manufacturing larger quantities of chocolate, but, at the same time, while manufacturers were asked to ensure the quality of their products, anxiety grew over the unhealthy chemicals and fillers that, when blended during the production, prevailed over the natural medical benefits of chocolate [3].

Alternative recipes had been developed also in the past, substituting cocoa with other cheaper ingredients, that, however, offered the same aspect. An example was the work of Doctor Saverio Manetti who, in 1765, printed a book, where he suggested very economical stratagems, to cope with a recession due to poor harvest: people who could not afford to buy chocolate, could use roasted flour, milk, sugar and egg yolks, in order to obtain a beverage, which at least looked like chocolate [33].

The addition of milk to chocolate firs occurred at the end of the 17th century, but Nestlè preparation based on condensed powdered milk by evaporation started in 1867, completely changing the taste of chocolate. Given the rise of milk chocolate products, it was also the purity of sugar and milk that came under inquiry, as overly processed chocolate contains added sugar and saturated fatty acid, which offset cocoa health benefits. On the one hand, milk chocolate had become a sort of highly nourishing food, but on the other hand, sugar and milk had paved the way to a negative perception of chocolate itself. During the 20th century, chocolate was demonized as a high-fat, high-calorie food, bearing the burden, over time, of a negative valence, generating a *topos* in medical and non-medical literature, in which it was associated to obesity, dental problems, unhealthy regimen of life and so forth [3].

## 6. Conclusions: Chocolate as Medi-Food

During the 20th century, the concept of chocolate being good for the diet overcame its concept as medicine, above all, when it was introduced as an essential constitutional drink or snack in soldier rations. Ancel Keys (1904–2004), a University of Minnesota public health scientist who proposed that saturated fat was a major cause of heart disease and championed the benefits of the Mediterranean diet, had designed a lightweight, but nutritionally robust ration for paratroops, the K Ration, named after him, which was originally made up of hard biscuits, dry sausage, hard candy and chocolate. The K rations were consumed by millions of soldiers in World War II [34].

The concept of chocolate as food seemed to have overcome its concept as medicine. The rehabilitation of chocolate, also from a medical point of view, occurred only in recent times, when biomedical science began to search for evidence of its benefits, just as it did for other pharmaceutical products. In the late 1900s, claims of chocolate benefits focused upon its richness in carbohydrates, fat and phytonutrient flavonoids, confirming the benefits of dark chocolate in cardiovascular diseases, gastrointestinal and respiratory disorders and mental health. However, chocolate consumption may also proffer a host of other health benefits, due to its antioxidant and anti-inflammatory properties, which are thought to be responsible for much of the health benefits ascribed to chocolate consumption [35,36].

Increasing data show that chocolate consumption improves brain function, as countries with more chocolate consumers apparently produce significantly more Nobel laureates, possibly through enhanced cognition [37].

Chocolate as food, then, or as medicament? Surely, chocolate, as a functional food, that recognizes and generates interesting physiological effects, is likely to promote or maintain health; hence, chocolate as “medi-food”, which exalts its nutritional functions and its therapeutic abilities [4].

Chocolate, therefore, positively recaptured the attention of international scientific magazines, restoring that value that Linnaeus himself credited chocolate with, calling the generous plant, *Theobroma Cacao*, food of the gods [1].
